# Status and associated factors of birth registration in selected districts of Tigray region, Ethiopia

**DOI:** 10.1186/s12914-020-00235-x

**Published:** 2020-07-29

**Authors:** Shishay Tadesse Abay, Ataklti Gebreyesus Gebre-egziabher

**Affiliations:** 1 Center for Population and Development, Institute of Population Studies, Mekelle University, P.O. Box 231, Mekelle, Ethiopia; 2 Center for Population and Development, Institute of Population Studies, Mekelle University, Mekelle, Ethiopia

**Keywords:** Birth registration, Birth certificate, Tigray region, Vital statistics

## Abstract

**Background:**

Birth registration establishes the existence of a child under law and provides the foundation for ensuring many of his/her rights. Despite its significance, a continuous, effective and comprehensive birth registration system has not been established in Ethiopia until the recent past. This paper examines the status of child’s birth registration and its associated factors in selected districts of Tigray Region, Ethiopia.

**Method:**

A community-based cross-sectional study was conducted from April to May 2018 among 383 randomly selected mothers who had given birth to at least one child since August 2016. A structured questionnaire was used to gather the quantitative data. Qualitative data were collected using key informant interviews and focus group discussions. To analyze the data, SPSS version 20 was used. Logistic regression analysis was employed to assess the association between dependent and independent variables.

**Results:**

Findings reveal that significant number of the respondents did not have knowhow about birth registration and its uses. As a result, only 117(30%) of them registered the birth of their children and secured certificates. Inaccessibility of the registrar offices, lack of relevant manpower and political will of the government were reported as major reasons for such a gap. Mother’s education was identified to be positively associated with the likelihood of a child being registered. Children born from mothers living in urban areas were found more likely to be registered compared to their rural counterpart [AOR = 1.46, 95% CI = 0.76, 2.76]. In light of Religion, children from the Muslim community had better opportunity for birth registration and owning birth certificate compared to children from Orthodox Christian parents. Compared to those who have possessed own birth certificates, the likelihood of mothers who did not possess own birth certificates to register the birth of their children was found lower by the factor of 86% [AOR = 0.14, 95%CI = 0.07, 0.26].

**Conclusion:**

Birth registration of a child and subsequent issuance of certificate should be pursued as a right issue. To make this a reality, extensive awareness raising programs that underscore the need for a birth registration and its significance for rural communities is needless to say critical.

## Background

Birth registration, the official recording of a child’s birth by a government agency is one of the most important events in a child’s life. As vividly stipulated in various human right instruments including the Convention on the Rights of the Child (UN, Article 7), all children have the right to the registration of a name, identity and birthdate [1].

Birth registration is fundamental because it establishes the existence of the child under law. However, apart from being the first legal acknowledgement that designates the child, registration of birth is more often than not vital to the realization of a number of rights and practical needs including provision of access to social services; protecting young people from under-age military service or conscription and securing the child’s right to a nationality at the time of birth or at a later stage. Additionally, it is essential for getting a passport, opening a bank account, obtaining credit, voting or finding employments [2]. In short, it is argued that birth registration is an essential step for an official recognition of a new member of society that can legitimately claim to all his/her rights and responsibilities as a full citizen [3]. Apart from these individual uses, particular records of birth and data derived from these records are important for evidence-based policymaking, service delivery and demographic analysis purposes [4].

Data on birth registration supplemented by other statistics such as death, divorce, and marriage are indispensable for setting targets, developing indicators and measurement of progress towards national and global human development targets such as Sustainable Development Goals (SDGs) [5]. The measurement of salient demographic indicators about levels of living such as life expectation at birth, infant, child, and maternal mortality rates are plainly or tacitly entwined with it [6–8].

A survey report disclosed by UNICEF shows that the percentage of self-reported birth registration under the age of five was 65% globally in the year 2013, which was improved to 71% in 2016 [9, 10]. Although this information gives the impression that affirmative progress has been witnessed globally, tremendous regional discrepancies have been observed across regions in the coverage of birth registration under the age of five. Such discrepancy was largely created by the poor system or absence of child registration in developing countries. For example, in May 2016, birth registration of children under the age of five was almost complete in advanced regions (99%), followed by Latin American and Caribbean (94%). On the contrary, birth registration of children under the same age was reported the least in South Asia (62%) and Sub-Saharan Africa (46%) regions [10]. This absence of birth registration is both a symptom and a cause of underdevelopment [11].

It is a cause to underdevelopment because the absence of complete birth registration has negative repercussions for children and a nation. For instance, it deprives a child of the privileges, rights and protection that a nation offers for its citizens and hampers overall national development process of a nation [12, 13]. It is said that social, cultural, economic and political factors, at both macro and micro levels have adversely affected birth registration in most parts of the developing world [14]. Factors like commitment of government, legislative framework of a country and absence of infrastructure that can support the logistical aspects of birth registration, especially in remote areas, the value that individuals and families give to birth registration, and lack of adequate knowledge of how to register a child are the bottlenecks associated with birth registration [9, 14]. Besides, background characteristics of parents like rural or urban residence, wealth and mother’s education are believed to affect the likelihood of birth registration [9].

Available sources in Ethiopia show that the formulation and issuance of relevant laws and policies for the enforcement of birth registration as part of a vital registration system dates back to the time of Emperor Menelik in the beginning of the twentieth century [13, 15]. Since then, a number of other legal and policy measures have been undertaken, mainly from the 1960s onward. The 1960 Civil Code of Ethiopia, the 1995 Constitution of the Federal Democratic Republic of Ethiopia (FDRE) and the Revised Family Law of Ethiopia issued in July 2000 recognize birth registration as one of the fundamental rights of children. Such decrees also set some provisions on how to execute birth registration as part of the wider vital registration system [16, 17]. However, despite all these official attempts, continuous, effective and comprehensive registration system has not been established in Ethiopia until the recent past [15].

Cognizant of the importance of birth registration and the problems associated with not doing so, the Government of Ethiopia has reorganized the legal framework and invigorated the national registration system. Proclamation on civil registration and national identification that compels for births to be registered within three months after birth was for example enacted by the Parliament in July 2012 [18]. However, Ethiopia launched throughout the country a permanent, compulsory and universal registration and certification of vital events such as birth, death, marriage and divorce on August 2016. Since then, complete and decentralized system of birth registration and officially issuing of certificates are underway in Ethiopia. The requirements for birth registration are identification card of the mother and father, name of the child and physical presence of both parents (unless for justified ground where both cannot be present) [18]. Given the limited empirical evidence on birth registration determinants, especially in Ethiopia, this study would contribute to addressing this gap by identifying factors associated with birth registration and certification status of a child. The study also examined the coverage of birth registration and possessing of certificates taking mothers who had given birth to at least one child since August 2016 as a proxy in selected districts of Tigray Region -Northern part of Ethiopia.

## Methods

### Study setting, design and period

A community-based cross-sectional study was conducted from April to May 2018. The study was conducted in four Woredas (districts) of Tigray Region, namely Shire-Endaslassie, Medebay-Zana, Aksum and La’elai-Maichew. Situated at the northern tip of Ethiopia, Tigray Regional State is one of the nine administrative regional states in the nation. According to the estimate made by Central Statistical Agency (CSA) of Ethiopia, the total population of the region in 2017 was 5.2 million of which, 1.4 million and 3.8 million people lived in urban and rural areas, respectively [19].

The target population of this study was all women who had given birth to at least one child since August 2016, the time when the official registration of birth and other vital events was commenced in Ethiopia. Mothers who had given birth prior to the specified time were intentionally excluded from the study. A single population proportion formula was used to determine the sample size for determining the percentage of registered children with birth certificates and associated factors. In sample size calculation, assumed 50% of registered children with birth certificates was taken because of lack of similar study conducted in Ethiopia. Accordingly, the sample size considered for this study is determined taking into account the standard score corresponding to 95% confidence interval, assumed proportion of registered children with certificates at 50% and the margin of error.

Accordingly, data were collected from 384 mothers. To select the sample size from the target population, systematic random sampling technique was used. At the beginning, from the seven administrative zones of the region, two zones (Central and North West) were taken using random sample. Following that, identification of the number of districts in each selected zone was made. Then, four districts (two from each zone) were selected randomly. The districts are Aksum town and La’elai-Maichew from central and Shire-Endaslassie and Medebay-Zana from North West Zones. Once the sample districts have been identified, eight Tabias (the smallest administrative unit) were considered randomly. Finally, systematic random sampling technique was employed to select the respondents. The list of eligible mothers was taken from the health extension workers of each Tabia. At the time of data collection, the age of the youngest participant of this research was 18 years. To get representative sample from each district, the study sites were initially stratified into urban and rural districts and final sample individuals were taken considering the number of mothers who had given birth to at least one child since August 2016 in each district.

An interviewer-administered structured questionnaire (see supplementary file_1) was used to gather the quantitative data. This instrument was deployed to collect data pertaining to demographic characteristics, awareness, and practice of birth registration in the study areas. Qualitative data were collected using Key Informant Interviews (KII) and Focus Group Discussions (FGDs). An interview guide was developed to collect qualitative data from the key-informants (Supplementary file_2). Four key informant interviews and two FGDs were held. The key informants interviewed were officers of civil status assigned to register vital events and issue certificates including birth in each study area. The participants of the FGDs were deemed to represent the local community and hence district officials, religious leaders, leaders of community based organizations such as *Idir*, women and youth associations and parents were included.

All returned questionnaire were checked for completeness and consistency of responses manually. After such cleaning, the items were coded and entered into Statistical Package for Social Sciences (SPSS) version 20, and the data entered were checked on normality. Analysis of the data was done using univariate, bivariate and multivariate analyses. Univariate analyses such as frequency and summary measures were used to depict the background attributes of the respondents, whereas bivariate analysis was used to examine the relationship between whether mothers have registered the birth of their children and get birth certificate or not with socio-economic covariates. To identify the factors that influence mothers’ propensity to register and get birth certificates for their children, logistic regression model was applied. Odds ratios at 95% Confidence Interval (CI) were also computed and reported. To see the relationship between the outcome variable and each predictor variable, at the outset binary logistic regression was used. Predictor variables that turned out significant (P ≤ 0.05) at this level were considered for further analysis. The set of predictor variables considered were identified from previously conducted researches in different settings. The dependent variable in this study- registration and possessing of birth certificate of a child or not, is a binary variable which took a value one if mother did register the birth of her child and got birth certificate, zero otherwise. At the time of data collection, mothers who did register the birth of their children were asked to show the certificate; because in Ethiopia, the issuance of a birth certificate immediately follows birth registration.

## Results

### Background characteristics and reproductive history of respondents

From the 384 eligible mothers, 383 respondents (99.7%) fully responded to the interviewer administered questionnaire. The age composition of the respondents shows that 139 (36.3%) of them were 24–28 years old, followed by 29–33 years 89 (23.2%) and less than or equal to 23 years 86(22.3%). The remaining 69(18%) were aged higher than 34 years. The mean age of the women was 28 ± 5.6 years. Out of the total respondents, 239 (62.4%) were living in urban areas at the time of the survey and the remaining 144(37.6%) were rural residents. With regard to the marital status of the respondents, the majority 291(76%) were currently married, while 39(10.2%) were never married. The predominant religion was Orthodox Christianity for 351 (91.6%) respondents, followed by Muslim 32 (8.4%).

The educational level of the respondents indicates that 142 (37%) and 119 (31%) of them have completed primary (1–8) and secondary (9–10) education, respectively. Surprisingly, 76(20%) of the respondents have not received formal education. Only 46 (12%) have completed preparatory and above level of education. The finding further uncovers that majority of the respondents were self-employed mainly in agriculture and trade which account 93 (24.3%) and 75(19.6%), respectively. The respondents who relied on agriculture for their means of livelihood are underemployed because of the seasonal nature of the activities. Moreover, 43 (11.2%) of the respondents were housewives virtually responsible for family care and household chores. The proportion for the unemployed was 22.2%. The remaining were government employees, hired in private organizations and daily laborers (Table 1).

**Table 1:** Socio-demographic and economic characteristics of the participants; Tigray Region, Ethiopia, May, 2018

Variable (*N* = 383)	Frequency	Percent
Age		
≤ 23	86	22.5
24–28	139	36.3
29–33	89	23.2
≥ 34	69	18
*Place of Usual Residence*		
Urban	239	62.4
Rural	144	37.6
*Marital Status*		
Never married	39	10.2
Currently married	291	76
Others	53	13.8
*Religion*		
Orthodox	351	91.6
Muslim	32	8.4
*Educational Level*		
Illiterate	76	19.8
Primary completed (1–8)	142	37.1
Secondary completed (9–10)	119	31.1
Preparatory completed (11–12)	19	5.0
Diploma and Above	27	7.0
*Primary occupation*		
Government Employee	42	10.9
Hired in Private Organization	22	5.7
Trader	75	19.6
Daily laborer	23	6.1
Agriculture	93	24.3
Housewife	43	11.2
Unemployed	85	22.2

The reproductive history of the respondents unveils that 241(63%) of the mothers have given birth to less than or equal to two children, and 125 (32.7%) of them had 3–5 children. The average number of birth was 2.4 ± 1.4 children. Here, mothers were inquired to report the birth order of their last child (latest birth). Accordingly, the result pointed out that 121(31.6%) and 120(31.3%) of the respondents reported that the birth order of their last child was 1st and 2nd, respectively. The age at first birth of the mothers indicated that 181(47.2%) of them gave birth to their first child between 20 and 24 years, and followed by less than or equal to 19 years which accounted 111(29%). The median age at first birth was found to be 21 years. The result further showed that 84.6% of the mothers have visited the nearby health center at least once during pregnancy time for their last child to get antenatal care (ANC) service. It was also found that 86.7% of the mothers delivered their last child at health centers but the remaining gave birth at home, in the absence of professional support. The proportion of mothers who got postnatal care service was 308(80.4%) (Table 2).

**Table 2:** Reproductive history of the Respondents; Tigray Region, Ethiopia, May, 2018

Variables	Frequency	Percent
Number of Children		
≤ 2	241	62.9
3–5	125	32.7
≥ 6	17	4.4
Birth order of the last child		
1st	121	31.6
2nd	120	31.3
3rd	66	17.2
4th	43	11.3
5th	18	4.7
6th	15	3.9
Age at first birth of Mothers		
≤ 19	111	29
20–24	181	47.2
25–29	80	20.9
≥ 30	11	2.9
ANC visit during pregnancy for the last Child		
Yes	324	84.6
No	59	15.4
Postnatal Care Service		
Yes	308	80.4
No	75	19.6
Place of Delivery of the last child		
At Home	51	13.3
At Health center	332	86.7

### Knowledge about birth registration and certification

Table 3 presents respondents’ awareness and knowledge about birth registration. It has been found out that from the total respondents considered, only 172 (45%) of them have ever heard about birth registration. In this regard, while 55 (31.9%) of the respondents indicated that the main source of such information was media, 54 (31.5%) of them said they got it from health institutions. In addition, the data indicated that families and neighbors were important sources of information concerning birth registration. Out of the respondents who have ever heard about birth registration, 93 (54.1%) of them knew the legal time when to register the birth of a child and secure birth certificate, whereas 79(45.9%) did not know when to register the birth of a child. Again, of the respondents who have ever heard about birth registration, 123 (71.5%) knew where to register the birth of a child and get certificate including the specific concerned office in charge of the task. Notwithstanding, only 92(53.5%) of the respondents had knowhow about the uses of birth registration and possession of certificate. The remaining 80(46.5%) knew nothing about the uses of birth registration and certification. The question here may be what were the yardsticks used to see respondents’ knowledge or information about birth registration?

**Table 3:** Responses of the participants to the knowledge related information on birth registration and certification of a child, Tigray Region, Ethiopia, May, 2018

Have you ever heard about Birth Registration (*N =* 383)	Number	Percent
Yes	172	44.9
No	211	55.1
Main source of information for those who ever heard (*N =* 172)		
Medias	55	31.9
Training or Meeting on birth registration	24	13.9
Families and/or neighbors	39	22.7
Health institutions	54	31.5
Do you know the legal time when to register the birth of a child (*N =* 172)		
Within three months after live birth	93	54.1
After four months following live birth	30	17.4
I do not know	49	28.5
Do you know where to get birth certificate (*N =* 172)		
Yes	123	71.5
No	49	28.5
Do you know some uses of birth registration (*N =* 172)		
Yes	92	53.5
No	80	46.5
Summary index of knowledge (*N =* 172)		
Knowledgeable	99	57.6
Not knowledgeable	73	42.4

To ascertain the aggregate level of knowledge of the respondents about birth registration, a series of knowledge related questions were asked. The knowledge related questions asked were ‘have you ever heard about birth registration? Do you know the legal time when to register the birth of a child and secure birth certificate? Do you know where to register a child and get birth certificate and do you know some uses of birth registration and having certificate?’ Consequently, respondents who answered 75% of the knowledge related questions correctly were categorized as knowledgeable, yet those who responded less than the specified value were labeled as unknowledgeable. As a result, the overall summary index for knowledge about birth registration revealed that 99 (57.6%) of the respondents were knowledgeable while 73(42.4%) of them were otherwise (Table 3).

### Status of birth registration and certification

This study has shown that, although 172 (44.9%) mothers had heard or been aware of birth registration services, only 117(30%) of them did register their children at birth and possessed birth certificates. The majority, 266(70%), did not register their last child and had no birth certificates at all. From the total respondents who registered and secured birth certificates for their last child, 71 (60.7%) registered their children and acquired certificates within 90 days (legal time) following the date of live births whereas the remaining 39.3% did it after 3 months. Information from the FGDs and interviews demonstrated that registration of birth as an act of recording and recognizing the birth of a child by the government was largely unknown at the level of community. During the interview with mothers, we frequently witnessed the tendency to equate birth registration and certification with non-birth registration documents or cards acquired from churches and health institutions. More specifically, a significant number of the respondents considered cards or pieces of papers issued for vaccination as birth registration and certification evidences. Whenever we asked mothers to show us the certificate, they proudly revealed us cards issued by health institutions for vaccination purpose or cards acquired from churches for baptism because churches record the birth of a child when it is baptized. Nevertheless, these cards are limited in coverage and quality when they were viewed from the criteria of what birth registration document is supposed to include. Moreover, absence of coordination between the registrar offices and other concerned bodies like health institutions and police, shortage of infrastructure, inaccessibility of the registrar office and lack of commitment on the side of government organs were repeatedly reported by the key informants as major predicaments for the implementation of the system.

### Associated factors of registration and certification of birth

Table 4 presents a summary of the results of associations between birth registration and certification status of children and the plausible independent variables. Accordingly, age of a mother, current residence of the respondents, religious affiliation, educational status, possession of own birth certificate of mothers, and place of delivery of the last child were the predictor variables found significantly associated to registration of the birth of a child and owing of certificate.

**Table 4:** Associated factors of birth registration among mothers who had given birth to at least one child since August 2016, Tigray Region, Ethiopia; May, 2018

Variable	Does the last birth registered?		Odds ratio (95% CI)			*P*-value
	Yes N (%)	No N (%)	Crude Odd Ratio (COR) (95% CI)	*P*-value	Adjusted Odd Ratio (AOR) (95% CI)	
Age						
≤ 23®	18 (20.9)	68(79.1)	1		1	
24–28	54(38.8)	85(61.2)	2.4(1.29, 4.47)	0.00	2.25(1.11, 4.53)	0.02
29–33	33(37.1)	56(62.9)	2.23(1.13, 4.37)	0.02	2.47(1.14, 5.39)	0.02
≥ 34	12(17.4)	57(82.6)	0.79(0.35, 1.79)	0.58	0.77(0.29, 1.99)	0.59
Current Residence						
Urban	89(37.2)	150(62.8)	2.46(1.51, 4.01)	0.00	1.46(0.76, 2.76)	0.02
Rural®	28(19.4)	116(80.6)	1		1	
Religion						
Orthodox®	102(29.1)	249(70.9)	1		1	
Muslim	15(46.9)	17(53.1)	2.15(1.04, 4.48)	0.04	2.42(1.06, 5.55)	0.04
Marital Status						
Never Married®	6(15.4)	33(84.6)	1		1	
Currently Married	104(35.7)	187(64.3)	3.06(1.24, 7.54)	0.02	2.02(0.73, 5.61)	0.17
Others	7(13.2)	46(86.8)	0.84(0.26, 2.72)	0.77	0.86(0.23, 3.26)	0.82
Educational status						
Illiterate®	11(14.5)	65(85.5)	1		1	
Primary (1–8)	49(34.5)	93(65.5	3.11(1.51, 6.44)	0.00	3.40(1.51, 7.65)	0.00
Secondary(9–10)	35(29.4)	84(70.6)	2.46(1.16, 5.22)	0.02	2.28(0.97, 5.30)	0.06
Preparatory & Above	22(47.8)	24(52.2)	5.41(2.29, 12.83)	0.00	4.35(1.60, 11.81)	0.00
ANC visit						
Yes	111(34.3)	213(65.7)	4.60(1.92, 11.04)	0.00	2.67(0.62, 11.37)	0.18
No®	6(10.2)	53(89.8)	1		1	
Place of Delivery						
At home®	7(13.7)	44(86.3)	1		1	
At health center	110(33.1)	222(66.9)	3.12(1.36, 7.14)	0.00	1.55(0.32, 7.45)	0.01
Does the mother has own birth certificate						
Yes®	49(62.8)	29(37.2)	1		1	
No	68(22.2)	237(77.7)	0.17(0.10,0.29)	0.00	0.14(0.07, 0.26)	0.00

**Keys**:®: Reference category

Age is one of the predictor variables found significant (*p <* 0.05) to determine mother’s probability to register the birth of her child. Compared to mothers aged ≤23 years, mothers aged between 24 and 28 years were 2 times more likely to register the birth of their children and get birth certificate [AOR = 2.25, 95%CI = 1.11, 4.53]. In addition to this, the likelihood of mothers aged between 29 and 33 years to register the birth of their children was 2.5 higher compared to mothers aged less than or equal to 23 years [AOR = 2.47, 95%CI = 1.14, 5.39]. Compared to the reference category (≤23 years), mothers aged greater than or equal to 34 years were less likely to register their children by a factor of 23%, though it was not found statistically significant.

Place of residence of the mothers was also found significantly related to their propensity to register the birth of their children. Urban residents were found 1.5 more likely to register the birth of their children compared to their rural counterparts [AOR = 1.45, 95%CI = 0.76, 2.76]. Variation in the likelihood of birth registration by religious affiliation of the mothers was also noticed. Compared to Orthodox Christianity followers, respondents with Muslim religious affiliation were found 2.4 times more likely to register the birth of their kids and secure certificates [AOR = 2.42, 95%CI = 1.06, 5.55].

Maternal education was also found to be highly significant to determine the proclivity of mothers to register their children in the study areas. Compared to those who had no formal education, mothers who have completed primary education were 3.4 times more likely to register and get birth certificate for their children [AOR = 3.40, 95%CI = 1.51, 7.65]; and those who have completed preparatory and above level of education were 4 times more likely to register and get birth certificate for their children than those who had no formal education [AOR = 4.35, 95%CI = 1.60, 11.81]. Likewise, although it is not statistically significant, the likelihood of registering the birth of children was 2.3 times higher for mothers with secondary level of education compared to those who did not receive formal education [AOR = 2.28, 95%CI = 0.97, 5.30].

The result of the study again revealed that the likelihood of mothers who did not possess own birth certificates to register the birth of their children was found lower by the factor of 86% [AOR = 0.14,95%CI = 0.07, 0.26] compared to those who have possessed own birth certificates. Moreover, those mothers who delivered at health institutions for their last births were 1.6 times more likely to register the birth of their children than those who delivered at home [AOR = 1.55, 95%CI = 0.32, 7.45].

## Discussion

This study explored birth registration in selected districts of Tigray region, Ethiopia, with particular emphasis on the knowledge, status of birth registration and associated factors that affect the likelihood of a child gaining identity at birth among mothers who have given birth to at least one child since August 2016. This was pursued to tracking progress towards the implementation of birth registration in the region and find out the hurdles that impede the continuous, effective and comprehensive birth registration in the study areas.

The result reveals that majority (55.1%) of the respondents have never heard about birth registration. Even significant proportion of those who had information about it have not been well-informed because they did not know the legal time when to register a child and the legal office in charge of birth registration and related issues. As a result, 57.6% of the respondents who have ever heard about birth registration have knowledge about birth registration and the importance of birth registration and certificates for their child’s future, which is basically lower than the value (69.9%) found in urban communities of Southern Nigeria [20]. Overall, birth registration as a right and key to identity, citizenship and enjoyment and exercise of other rights is not something that most people in the community have ever thought about, particularly in remote rural areas where access to information and/or media outlets is scarce. Hence, the low level of awareness and knowhow of the respondents in particular and the community in general on the issue may be stemmed from dearth of undertaking persistent sensitization and massive awareness raising programs. And such low level of awareness seems to mirror with findings in other African countries. For example, a research conducted on urban communities in Nigeria indicate that majority of the respondents were not aware of the existence of government office in charge of birth registration and only about one-third (32.5%) of them knew when to request for a birth certificate [20].

The result on the level of birth registration depicts that only 30% of the mothers who had given birth to at least one child since August 2016 had registered the birth of their children and secured certificate for it. Here, the legal framework enacted in Ethiopia to execute birth registration and other vital events needless to say stipulates that births shall be registered within 90 days of the occurrence of the event [18]. However, in our study, it was found that only 60.7% of the mothers registered the birth of their children in the specified time while the remaining did it lately after 90 days of delivery which is designated as *late registration* by the decree. In this regard, it is possible to say that the percentage of registered children and received birth certificate is low in the study areas compared to results in Ghana (52.4%) [14], in three jurisdictions of Indonesia (46%) [21]. Nevertheless, our findings indicate that birth registration in the study areas is slightly higher than the Eastern & Southern Africa regional average birth certificate coverage (26.9%) [22]. The general lack of awareness among parents and guardians of the need for and importance of birth registration and certificates for their child’s future, inaccessibility of the registrar office (distance between residence and the office), lack of relevant manpower and insufficient infrastructure were reported as major reasons for such a gap. Besides, it was unanimously explicated that lack of political will of the government is one of the basic problems in executing the system in the region. Absence of registrar officer that deals exclusively with birth registration and other vital events at Tabia level, and lack of infrastructure and trained manpower are some of the manifestations signaling the low priority given to the system. This result is consistent with study conducted in Sudan. The value that families and individuals give to birth registration, the existence of insufficient infrastructure, and the number of barriers families encounter during registration were reported as major factors that impeded birth registration in the country [22].

The results of multivariate analysis demonstrate that Muslim children have higher chances of registering and acquiring birth certificate. This might be due to the fact that in the history of Ethiopia, there has not been practical official registration of births and other vital events despite the enactment of piece of laws and legal frameworks. In this regard, readers have to notice that the dominant group is follower of Orthodox Christianity. The dogma of the church orders that a baby irrespective of sex must be baptized in order to be counted as member of the Christian community. Hence, Christian followers acquire a card or document for their children from their church during the baptism day. This card seems official birth certificate. Owing to this deeply rooted practice, parents affiliated to Christian Religion have the tendency to consider the card obtained from the churches as the official birth certificate. This in turn resulted in abandoning the official registration of their children even official recording of births is in place. However, the flaw of the document given by the church is that it is not official because it fails to recognize and protect personal, and socio-economic and political rights. This needless to say created disparity in the level of official birth registration among the Christian and Muslim Religious followers. This finding is not typical to the study area, however. It is so because similar research findings were revealed in some African countries like Ghana where children from Muslim households had higher likelihood of being registered compared to their Christian counterparts [14].

Education is also found to have its impact on birth registration. The finding that maternal education improves the likelihood of children being registered is not something unanticipated as it is consistent with empirical evidences and general perceptions. For instance, empirical studies in different African countries such as Ghana [14], Laos [23], Zambia [24], and other regions [25] indicate that child registration and level of maternal education have relation. More precisely, well-educated women are more likely to be acquainted with the importance of birth registration for their children and beyond because educational status has an impact on mothers’ level of awareness about birth registration. Accordingly, this study has shown that mothers who had adequate knowledge about birth registration and its uses are more likely to register their children than their counterparts. Again, place of residence was found to have its own impact on child birth registration. In other words, the study unveils widespread variations in child registration by place of residence of mothers in that urban children are more likely to be registered than their rural counterparts. This is perhaps due to demand and supply differentials between urban and rural areas. It could also be owing to the fact that urban communities are more aware of the uses of identity registration and procedures on birth registration compared to rural communities.

Maternal age was also found as a significant factor for birth registration in the study areas. As indicated in the multivariate analysis, the probability to register birth of their children was found higher among mothers aged 24–28 years and 29–33 years compared to mothers aged less than 24 years. This result is also consistent with findings reported in Laos [23]. This is because unmarried women who give birth out of wedlock do not want to register the birth of their babies and officially publicize their fathers as premarital birth is traditionally deemed as a taboo in the study areas.

### Limitation of the study

The data were collected from mothers who have given live births for at least one child since August 2016 and whose children were alive at the time of data collection. However, mothers who had given live births for their last children but whose children were not alive at the time of data collection were not part of the study. Thus, birth registration may have been over or under reported in this study. The prevalence of the outcome found is relatively high (30%), which could be derived from information bias. Consequently, there might be an overestimation of the relative risk in the model. Moreover, this result cannot be generalized to the entire population of Ethiopia, but only to the population of the study region (Tigray Region).

## Conclusion

Registration of birth and acquiring certificate promotes not only the protection of fundamental rights of children but also the provision of educational, health, social security, and other social and economic services. However, findings from this study demonstrate that majority of the respondents have never heard about birth registration, significant numbers do not have knowhow about the procedures of birth registration including the legal time when to register their children and the importance of birth registration and certificates for their child’s future.

To improve the status of birth registration to the desired level, therefore, organizations working at local level should create awareness among community members. In this regard, organizations engaged in activities related to schools and health centers should play a dominant role in creating opportunity to encourage and implement birth registration. Such organizations should create clear and expansive awareness raising campaigns on the part of the community regarding the difference between official birth registration and documents obtained from churches and other institutions. This can encourage mothers to register their children with reasons. Furthermore, the government has to prioritize birth registration. Intervention programs initiated by the government and other stakeholders should focus on rural areas, an uneducated segment of the community. Doing so is vital because findings of this study seem to suggest that birth registration is more of a privilege for a small number of children whose mothers are educated and live in urban areas. Finally, further nationwide comprehensive research is required to ascertain the institutional arrangements, effects of low birth registration and intervention programs.

## Supplementary information

**Additional file 1 :** Survey questionnaire used to assess the status and associated factors of birth registration in selected districts of Tigray Region, Ethiopia, may, 2018.

**Additional file 2 :** An interview guide used to assess the status and associated factors of birth registration in selected districts of Tigray Region, Ethiopia, may, 2018.

## Data Availability

The datasets used and/or analyzed during the current study are available from the corresponding author on reasonable requests.

## Abbreviations

ANC: Antenatal Care Services
AOR: Adjusted odds ratios
CI: Confidence interval
COR: Crude odds ratios
CSA: Central Statistical Agency
FDRE: Federal Democratic Republic of Ethiopia
FGD: Focus Group Discussion
KII: Key informant interview
SDGs: Sustainable Development Goals
SPSS: Statistical Package for Social Science
