# Supplementary material for: Status and associated factors of birth registration in selected districts of Tigray region, Ethiopia
Source: BMC Int Health Hum Rights. 2020 Jul 29;20:20. doi: 10.1186/s12914-020-00235-x (PMC7388520; doi:10.1186/s12914-020-00235-x)
Supplement: Supplementary file 1 — Additional file 1 : Survey questionnaire used to assess the status and associated factors of birth registration in selected districts of Tigray Region, Ethiopia, may, 2018. [file 12914_2020_235_MOESM1_ESM.docx]

**Mekelle University**

**Institute of Population Studies**

**Survey Questionnaire**

**Dear Respondents!**

The researchers are a faculty member of Institute of Population Studies, Mekelle University. Their names are ______________and___________. They are conducting a research study about birth registration in the district. A research study is a way to learn more about people. If you decide that you want to be part of this study, you will be asked to provide details regarding birth registration of your child. This process will take thirty minutes of your time. All information obtained in the questionnaire will be treated with confidentiality. Your participation in this study is voluntary. There are no foreseeable risks and direct benefits (incentives) to you for participating in this study. However, the benefits might be your awareness on the importance of registering your child. When the study completed, a report will be written about what was learned. The report will not include your name or that you were in the study.

You do not have to be in this study if you do not want to be. If you decide to stop after we begin or not answer questions that are personal, that’s okay too.

Do you agree to participate?

1. **General Information:**
2. Name of Zone:______________________Name of Woreda:_____________________
3. Name of Kebelle:____________________________
4. Date of Data collection: Starting date:____________End of data collection:__________
5. Name of date collector/interviewer:____________________________________
6. **Background characteristics of Respondents:**
7. Age of respondent in complete years:_______________________years
8. Place of current/usual residence: 1. Urban 2. Rural
9. Place of birth of respondent: 1. Urban 2. Rural
10. Religious affiliation of respondent:

1. Orthodox 2. Catholic 3. Protestant 4. Muslim 5. Others:________

1. Marital Status of respondent: 1. never married 2. Currently Married 3. Divorced

4. Widowed 5. Separated 6. Cohabited

1. Are you a household head? 1. Yes 2. No
2. How many number of family members do you Have? 1. Male ____ 2. Female______
3. What is the estimated average monthly expenditure of the household?__________birr/
4. Ethnicity of respondent: _____________________________________________
5. Educational Status of respondent (mother): 1. cannot read and write 2. Can read and write 3. Primary (grade 1-8) 4. Secondary & Preparatory (grade 9-12) 5. Certificate

6. Diploma Graduate 7. TVET graduate 8. First degree and above

1. Educational Status of the father: 1. cannot read and write 2. Can read and write

3. Primary (grade 1-8) 4. Secondary & Preparatory (grade 9-12) 5. Certificate

6. Diploma Graduate 7. TVET graduate 8. First degree and above

1. What is your current employment status? 1. Employed 2. Unemployed 3. Student
2. If you are currently employed, what is your main occupation or means of livelihood?

1. Government employee 2. Hired in private sector 3. Self-employed (trade)

4. Daily laborer 5. Agriculture/herding 6. Others (specify):___________

1. What is your estimated average income per month? _______________Birr per month
2. If your means of livelihood is crop production or mixed farming, what is the size of the farm land in hectare? _________________hectare.
3. **Reproductive History of the mother**
4. How many number of children do you have? _______________children
5. What is the birth order of your last child? __________________
6. How old were you when you give birth for your first child?_________________years
7. Sex of your last child: 1. Male 2. Female
8. When did you give birth for your last child? Month____________year_______
9. Have you received antenatal care service when you were pregnant for your last child at health centers? 1. Yes 2. No
10. Have you received postnatal care service following your delivery of your last child?
11. Yes 2. No
12. Where did you deliver your last child? 1. at home 2. At health center 3.other_____
13. Does the biological father of the child present in the household? 1. Yes 2. No
14. Age of Your last child? Month____________________year_____________________
15. Do you have your own birth certificate? 1. Yes 2. No
16. Does your last child get registered and has birth certificate? 1. Yes 2. No
17. If your last child is registered and has birth certificate, when did you registered your last child? 1. Within three months after birth 2. After three months following delivery
18. If your last child is registered and has birth certificate, from where did she/he get his/her birth certificate? 1. Religious institutions 2. From municipality

3. From Civil registry 4. From Health centers

1. If your last child is registered and has birth certificate, how far is the registration place from your residential area? ____________hours walk or ____________minutes’ walk
2. If your last child is not registered and has no birth certificate, why?
3. I do not know where to register my child
4. The registration office is too far from my home
5. I am not aware of the use of birth registration
6. Others (specify):_________________________________
7. Have you ever heard about birth registration? 1. Yes 2. No
8. If your answer for Q.12 is yes, how did you get the information?

1. From media (TV, Radio, Newspaper) 2. Social gathering

3. from training or meeting on birth registration 4.From family members or friends

5. Others (specify):__________________________________

1. When is the legal time set to register a new born Child? 1. Within three months after delivery 2. Four to six months 3. After one year 4. I don’t know
2. Do you know where the vital events registration office is? 1. Yes 2. No
3. Have you ever heard about Vital Events registration system? 1. Yes 2. No
4. If your answer for Q.21 yes, from which did you get the information?
5. From media (TV, Radio, Newspaper) 3. Social gathering
6. From training or meeting on birth registration 4. From family members or friends
7. Others (specify):__________________________________

*Thank You in advance!!*
