# Supplementary material for: Status and associated factors of birth registration in selected districts of Tigray region, Ethiopia
Source: BMC Int Health Hum Rights. 2020 Jul 29;20:20. doi: 10.1186/s12914-020-00235-x (PMC7388520; doi:10.1186/s12914-020-00235-x)
Supplement: Supplementary file 2 — Additional file 2 : An interview guide used to assess the status and associated factors of birth registration in selected districts of Tigray Region, Ethiopia, may, 2018. [file 12914_2020_235_MOESM2_ESM.docx]

***Mekelle University***

***Institute of Population Studies***

***Guidelines for key-informant interviews and Focus Group discussions***

***Dear interviewees and Discussants!***

Hello! My name is_______________, a researcher and faculty member of Institute of Population Studies, Mekelle University. We are conducting a research to examine the status and associated factor of birth registration in your district. I believe that the research will help to show the level of birth registration in the area and recommend actions to be taken to improve the system. Hence, we are very much grateful for the sacrifice you pay to this end and you are kindly requested to participate in this study and provide information required from you. Your participation in this study is completely on voluntary bases and you have a right to refuse joining the discussion and interview. The interview and discussion will take an hour. All information obtained will be treated with confidentiality. Any reporting of data will be anonymous.

1. ***Interview Guideline for Key informants***
2. Are people aware of the presence of birth registration in the woreda?
3. How do you see the knowledge and perception of the people towards birth registration?
4. How many children’s birth is registered since the agency commenced registering?
5. What mechanisms are used to aware the public on birth registration?
6. What kind of information on birth registration you normally generate?
7. What is the actual geographical coverage of birth registration in the woreda?
8. What are the bottlenecks associated with birth registration in the woreda? And their possible solutions (making having birth certificate obligatory for children to get any service so that parents would register their kids to let them get the service.)
9. **Focus Group discussion guideline**
10. Have you ever heard about vital events registration system?
11. What were your sources of information?
12. Why you let your children to get registered and have birth certificate (probe: what do you think is the relevance of birth registration?
13. Do you know where to register birth of your child and secure certificate?
14. What challenges did you face when you try to register your children?
15. What should be done to improve the completeness of birth registration?
